# The association of knowledge, attitudes and behaviors related to salt with 24-h urinary sodium, potassium excretion and hypertensive status

**DOI:** 10.1038/s41598-022-18087-x

**Published:** 2022-08-16

**Authors:** Xiaofu Du, Le Fang, Jianwei Xu, Xiangyu Chen, Yamin Bai, Jing Wu, Lin Wu, Jieming Zhong

**Affiliations:** 1 Department of Chronic Disease Prevention and Control, Zhejiang Provincial Center for Disease Control and Prevention, No. 3399 Binsheng Road, Hangzhou, 310051 China; 2 National Center for Chronic and Noncommunicable Disease Control and Prevention, Chinese Center for Disease Control and Prevention, 27 Nanwei Road, Beijing, 100050 China; 3 Department of Medical College, Jinhua Polytechnic, No. 888 Haitang West Road, JinHua, 321017 China

**Keywords:** Public health, Nutrition, Health care

## Abstract

To understand the association between sodium and potassium consumption levels, hypertension and knowledge, attitudes and behaviors (KAB) toward salt and the commitment to effective sodium reduction and potassium supplementation to achieve the purpose of suppressing hypertension. A stratified multistage random sampling method was used to obtain a representative provincial sample of 7512 residents aged 18–69 years through a cross-sectional survey by the Salt Reduction and Hypertension Prevention Project (SRHPP) in Zhejiang Province of China in 2017–2018. A screening including demographic, anthropometric, salt-related KAB and physical measurements was implemented, and 24-h urine of approximately 1/5 of the participants was collected and tested. The mean age was 44.8 years, 50.1% were women, 44.0% lived in urban areas, and hypertension or prehypertension accounted for approximately 35.0%. The mean 24-h urinary sodium and potassium excretion were 3848.5 (1661.1) mg/d and 1491.1 (710.9) mg/d, respectively. KAB in urban areas was generally more favorable than in rural areas, women were better than men, and the optimal blood pressure group was better than the other two groups (*P* < 0.05). However, the awareness and correct use rate of salt-restricted spoons, low-sodium salt and nutrition labeling were lower. A multivariable linear regression model indicated that KAB had a smaller effect on sodium (two indicators effective for promoting sodium reduction) and a greater effect on potassium (six indicators effective for promoting potassium supplementation) and mainly focused on knowledge and behavior indicators. A multivariable logistic regression model indicated that mastering more knowledge and taking active measures could effectively reduce the transition to hypertension, even if the individual was already in prehypertension. There is much room for improvement of salt-related KAB in the Chinese population. A clear association indicates that KAB can help to reduce sodium and supplement potassium, especially potassium, and help to suppress the development of hypertension. The role of beliefs in KAB should be fully valued and improved, similar to knowledge and behaviors. This study provides important evidence and insight into China’s efforts to meet the targets of salt reduction and hypertension prevention.

## Introduction

Public health is a top priority for the Chinese government, and the burden of noncommunicable diseases constitutes a major public health challenge in China and around the world. Increased blood pressure has become a major risk factor for the global burden of disease, and the resulting stroke and heart disease are the leading causes of death and DALYs at the national level in China^1^. Higher levels of salt intake cause increased blood pressure, especially in salt-sensitive individuals, a major risk factor for cardiovascular diseases (CVDs)^2^. The average daily salt intake in China and around the world is 10.5 g/d^3^ and 9–12 g/d^4,5^, respectively, which are far higher than the recommended amount by the Chinese Dietary Guidelines (less than 6 g/d)^6^ and the World Health Organization (less than 5 g/d)^7^. If salt consumption falls below the recommended amount, it will have a major impact on blood pressure and CVDs, preventing approximately 2.5 million deaths from stroke and heart disease worldwide every year^8^.

Furthermore, due to the low intake of vegetables and fruits and a lack of potassium supplementation habits, the potassium intake of the Chinese population is 1.42 g/d, less than half of the WHO recommended amount of > 3.5 g/day^9^. A higher potassium level (within a certain range) is conducive to the reduction of blood pressure and the prevention and treatment of CVDs^10–13^. Increased potassium intake will inhibit the function of the sodium–potassium pump and inhibit the release of renin, increasing the excretion of water and thereby reducing blood pressure.

Modeling studies and randomized trials (TOHP I and TOHP II) have emphasized that people would derive substantial health gains from the implementation of salt-reduction policies^14,15^. Therefore, reducing sodium intake and increasing potassium intake in the general population has been considered a cost-effective action that should be maintained and expanded to save lives, prevent diseases and reduce costs^16,17^. Since combining a moderate sodium intake (3–5 g/d) with a high potassium intake (> 3.5 g/d) is associated with the lowest risk of mortality and cardiovascular events, the WHO recommends sodium intake < 2.0 g/d and potassium intake > 3.5 g/d, respectively, which is a key strategy to lower blood pressure in China^18^.

Compared to Western countries where processed foods are the main source of salt, salt in Asian countries such as China are mainly sourced from home cooking^19^. Excessive salt intake is particularly prominent in many low- and middle-income countries^5^. Factors such as society, culture, economy, and dietary traditions are the external environment that dominates dietary behavior, and it is difficult to change in a short period of time. Population age, education level and average income are the main internal determinants of dietary behavior, and it takes a long time to change ingrained dietary habits^20^. However, the population’s knowledge, attitudes and behaviors are believed to affect salt consumption and are considered to be adjustable and controllable intermediate factors in a short time^21^. Therefore, targeting consumers to popularize salt-related knowledge, increase salt-reduction awareness, improve salt-reduction skills, and provide environmental and tool support to guide salt-reduction behavior are common measures for salt-reduction intervention^22^. However, studies to determine which core knowledge and skills are necessary and effective are scare but urgently needed by researchers and policy-makers. The purpose of this study was to describe the association between salt consumption levels, hypertensive status and salt-related knowledge, attitudes and behaviors (KAB), to identify KAB that are helpful for sodium reduction and potassium supplementation in order to improve the current status of hypertension in the population and to enhance the effectiveness of salt reduction efforts based on health promotion and education to maximize the health benefits.

## Methods

### Design

Cross-sectional survey data were analyzed from the Salt Reduction and Hypertension Prevention Project (SRHPP) in Zhejiang Province of China in 2017–2018 to study hypertension and salt intake and to provide salt reduction strategies. The required sample size to estimate the population prevalence of hypertension was 7500, and 1500 were used for salt intake estimation^23^. A complex, 4-stage stratified random sampling method was used to select the participants without disability and mental disorders living in the selected areas for 6–12 months before the investigation. Briefly, 5 project points, including 2 rural areas and 3 urban areas divided according to household registration, were distributed in the east, northeast, central, midwest, and south of Zhejiang Province to achieve balance and representativeness. For the geographic distribution map, see Supplemental Fig. S1. Eventually, 7512 respondents participated in the project with a complete database, among which 1496 respondents had complete 24-h urine collected to assess the overall sodium intake. The specific process of sample size calculation and sample selection has been published elsewhere^23^. SRHPP was approved by the Zhejiang Provincial Center for Disease Control and Prevention (CDC) ethics review board, and the researchers obtained informed consent from all participants. For the questionnaire surveys, physical examinations and biological specimen testing, all methods were performed in accordance with the relevant guidelines and regulations.

The investigators in this study underwent strict and uniform technical training and conducted a closed survey of the participants in a face-to-face manner. The demographic characteristics, history of hypertension, diabetes and cardiovascular disease, as well as the living habits of smoking, drinking, and physical activity of the participants, were collected through questionnaire surveys. This information was used as an adjustment variable for the association study.

Salt-related KAB of the participants was considered the main exposure variable for the study, which was obtained through a questionnaire survey (see Supplemental Table S1). The questionnaire design referred to the World Health Organization/Pan American Health Organization (WHO/PAHO) protocol for population-level sodium determination and was adapted from the related literature and through consultations with public health practitioners and experts^24–26^. Twenty-four KAB questions were set into 3 categories, mainly knowledge, belief and behavior, all related to salt and hypertension. The answer that best fit their situation or was considered right was chosen by the participants and recorded on the tablet by the investigator. During the data analysis, we classified and adjusted the multiple-choice question option into two categories of "yes" or "no". If the participant selected at least one correct option, it was judged as "yes"; otherwise, it was considered "no". The purpose of this approach was to facilitate analysis and to draw simple and effective conclusions. Among these, we set up a series of questions on the same topic, including the awareness, adoption, proper use and nonadoption reasons for salt-reduction tools (salt-restriction spoons, low-sodium salt, nutrition labeling, etc.), which are described in detail elsewhere^27^. Interpretation of survey questions is given first, and then the respondents provide their own answers, and finally the investigators check the corresponding options.

### Main outcomes and measures

The 24-h urine was used to calculate the sodium and potassium excretion of the participants within 24 h. During the investigation, a leaflet with explanations along with the necessary equipment (a 3 L standard urine collection container) was given to 1,572 participants, and the 24-h urine retention method was applied^28^. At the urine collection site, the 24-h urine volume was measured, and the beginning and end times of urine collection were recorded to assess the completeness of the 24-h urine collection. The urine specimens were considered incomplete if the length of collection time was less than 22 h, the total urine volume was less than 500 ml, or the 24-h urinary creatinine excretion was ± 2 standard deviations outside of the sex-specific mean^29,30^. Five milliliters of 24-h urine was transferred to a cryotube with a pipette and transported to the laboratory (KingMed Diagnostics Laboratory Inc., Hangzhou, China) using a cold chain for electrolyte testing. Urinary sodium and potassium were measured with the ion-selecting electrode method by an automatic biochemical analyzer (C16000, Abbott Corp., America), and urinary creatinine was measured with the picric acid method by an automatic biochemical analyzer (C501, Roche Cob as Corp., Switzerland). The test concentration was multiplied by the 24-h urine volume to obtain the excretion content of each indicator. Among the participants selected for 24-h urine collection, 1496 (95% of 1572) returned a complete specimen.

Brachial systolic blood pressure (SBP) and diastolic blood pressure (DBP) were measured according to internationally accepted measurement methods and quality control specifications with a calibrated electronic sphygmomanometer (HEM-7071, Omron Corp., Japan). Participants sat quietly, their upper arm was at the same level as the heart, and the lower edge of the cuff was 2.5 cm above the elbow. The measurement was carried out 3 times in succession at intervals of 1 min, and the average value of the measurement results was taken. The participants were divided into 3 categories based on blood pressure and antihypertensive medication usage. Hypertension was defined as a mean SBP ≥ 140 mmHg and/or a mean DBP ≥ 90 mmHg and/or the self-reported use of antihypertensive medication within 2 weeks. Prehypertension was defined as a mean SBP of 120–139 mm Hg or a mean DBP of 80–89 mm Hg, and an optimal blood pressure was defined as a mean SBP < 120 mm Hg and a mean DBP < 80 mm Hg without antihypertensive medication^31^.

### Statistical analysis

The baseline characteristics of the participants were summarized as proportions and means (SD, standard deviation). ANOVA for continuous variables and the Kruskal–Wallis test for categorical variables were used to compare the demographic and health characteristics across hypertensive status categories. Descriptive statistics were conducted for KAB-related variables, and the Mann–Whitney U test or Kruskal–Wallis test was used for comparisons within subgroups defined by sex, household registration type and hypertensive status category. Multivariable linear regression was used to assess the associations of KAB-related variables with 24-h urinary sodium excretion (mg) and potassium excretion (mg). For hypertensive status, multivariable logistic regression was used to assess the associations with the odds of hypertension from each KAB-related variable, comparing the adjusted odds for optimal blood pressure and prehypertension versus hypertension, with an approach similar to that used in linear regression models. The preliminary model was adjusted for age, sex and ethnicity. The fully adjusted model additionally included BMI, education, history of CVDs, diabetes mellitus, chronic kidney disease, smoking status, alcohol use status and physical activity. Statistical analyses were performed with SPSS for Windows (Version 26, SPSS Inc., Chicago, IL). *P* values < 0.05 were deemed significant.

## Results

### Study subjects

In our study, the average age of the participants was 44.8 (14.0) years old, 50.1% were women, 98.0% were of Han ethnicity, and over 2/3 were classified as hypertensive (35.3%) or prehypertensive (35.5%). The prevalence of hypertension was higher among men than women and higher in rural areas than in urban areas. Participants with hypertension generally had a higher age, lower education attainment, greater BMI, and higher smoking and drinking rates, as well as a significantly higher proportion of diabetes mellitus, CVDs, and chronic kidney disease than those with prehypertension or optimal blood pressure. In addition, the 24-h urinary sodium and potassium excretion of 1496 participants with urinary integrity were 3848.5 (1661.1) mg/d and 1491.1 (710.9) mg/d, respectively, and there was no significant difference across the hypertensive statuses. The amount of sodium excretion converted to salt consumption was 9.8 (4.2) g/d, which exceeded the WHO recommended maximum level of 5 g/d by 96.0% and exceeded the Chinese Nutrition Society recommended maximum level of 6 g/d by 63.3%. The relevant results are presented in Table 1.

**Table 1:** Participant characteristics by hypertensive status, SRHPP in Zhejiang Province of China in 2017–2018.

Characteristic^a^	All objects (n = 7512)	Optimal^b^ (n = 2194)	Prehypertensive^b^ (n = 2665)	Hypertensive^b^ (n = 2653)	*P* value for trend^c^
Age, year	44.8 (14.0)	37.1 (12.0)	42.9 (13.4)	53.1 (11.7)	< 0.001*
**Gender, %**					
Male	49.9	32.6	58.2	55.7	< 0.001*
Female	50.1	67.4	41.8	44.3	
**Ethnicity, %**					
Han	98.0	97.7	98.1	98.2	0.370
Others	1.9	2.3	1.8	1.8	
**Household registration type,%**					
Urban	44.0	41.9	42.9	46.7	0.002*
Rural	56.0	58.1	57.1	53.3	
**Education, %**					
< 9 years	30.4	18.3	25.5	45.2	< 0.001*
9–12 years	48.0	45.9	51.4	46.4	
> 12 years	21.6	35.8	23.0	8.4	
BMI (body mass index), kg/m^2^	23.9 (3.4)	22.3 (2.9)	23.7 (3.1)	25.4 (3.3)	< 0.001*
Stroke, %	0.8	0.0	0.3	2.0	< 0.001*
Coronary heart disease, %	1.0	0.1	0.3	2.3	< 0.001*
Diabetes mellitus, %	7.5	2.1	4.4	15.2	< 0.001*
Self-report kidney disease, %	0.3	0.0	0.0	0.8	< 0.001*
**Smoking status, %**					
Never smoked	71.5	81.4	67.3	67.6	< 0.001*
Former smoker	4.2	1.7	3.9	6.4	
Current smoker	24.3	16.9	28.7	26.0	
Alcohol use status, %	32.9	26.5	35.0	36.2	< 0.001*
Physical activity, %	40.2	39.3	40.6	40.6	0.604

	All objects (n = 1496)	Optimal (n = 456)	Prehypertensive (n = 507)	Hypertensive (n = 533)	*P* value for trend
24-h urinary sodium, mg/24 h	3848.5 (1661.1)	3886.1 (1694.8)	3854.2 (1647.5)	3810.9 (1647.1)	0.774
24-h urinary potassium, mg/24 h	1491.1 (710.9)	1518.1 (694.7)	1528.6 (775.4)	1432.5 (655.8)	0.058
Sodium-to-potassium ratio	4.9 (2.4)	4.8 (2.2)	4.9 (2.7)	5.0 (2.3)	0.314
24-h urinary creatinine, mg/24 h	1141.0 (545.7)	1124.2 (545.3)	1197.9 (567.2)	1101.1 (521.1)	0.012*
24-h urinary volume, ml/24 h	1449.3 (448.6)	1393.7 (446.3)	1451.9 (443.8)	1494.5 (450.7)	0.002*

^a^Samples sizes (n), means and prevalences are unweighted. Anti-hypertension medication use rate of hypertensive patients is 34.7%

^b^Hypertension defined as mean SBP ≥ 140 mm Hg, and/or mean DBP ≥ 90 mm Hg, and/or self-reported use of antihypertensive medication. Prehypertension defined as mean SBP 120–139 mm Hg or mean DBP 80–89 mm Hg. Optimal blood pressure was defined as mean SBP < 120 mm Hg and mean DBP < 80 mm Hg. Mean blood pressure was estimated from up to 3 readings during the physical examination in our baseline survey.

^c^Kruskal-Wallis test is used to compare the characteristics of different hypertensive status.

*P value for trend < 0.05.

### Knowledge, attitudes and behaviors toward salt and hypertension

Out of 7512 participants, although three-fourths of the participants knew at least one hazard (74.5%) and one risk factor (79.4%) for hypertension, the awareness rate of the diagnostic criteria for hypertension was only 53%. Consuming less salt can lower blood pressure, and the awareness of the hazards of high salt was higher among those who should consume less salt, 65.7%, 71.1% and 79.3%, respectively. The awareness of the recommended limit of salt of 6 g/d by the Chinese Nutrition Guideline was very low (38.9%). Most participants favored a low-salt diet (88.2%) and felt that it was necessary to promote this to the public (87.1%). Approximately half of the participants received publicity or education on a low-salt diet, and 69.0% claimed to have promoted low-salt diet knowledge to the surrounding population. Those who considered that a low-salt diet greatly affected the taste of food accounted for 11.6%, and those who described themselves as consuming “not much” or “moderate” salt accounted for 81.4%. When aware of having too much sodium intake, 78.0% planned to reduce salt, and 58.3% had taken the initiative to reduce their salt intake.

Three-quarters of the participants (75.1%) approved the nutrition labeling of prepackaged food, but only 1/5 paid attention to salt labels. Only 12.0% had used or were currently using salt-restriction spoons, along with 8.8% knowing how to use them correctly, and even fewer (5.6%) currently using them correctly. Less than one-third (30.0%) of participants had heard of low-sodium salt, followed by less than one-sixth (15.7%) who ate or were currently eating it.

These salt-related KABs were more favorable in urban areas than in rural areas, and most women were better than most men. The relevant results are presented in Table 2. For the hypertensive status categories, it was found that except for the diagnostic criteria of hypertension, the impact of a low-salt diet on the taste of foods, the approval of a low-salt diet and the awareness and adoption of a salt-restricted spoon, there were also significant differences for the other 18 variables of knowledge, attitudes and behaviors. The optimal blood pressure group was better than the other two groups (*P* < 0.05 for trend, Table 3).

**Table 2:** Knowledge, attitudes, and behaviors related to salt among Zhejiang residents, SRHPP 2017–2018: %.

	Total	Urban				Rural				*P*^b^
		Total	Male	Female	*P*^a^	Total	Male	Female	*P*^a^	
**Knowledge**										
Know the diagnostic criteria of hypertension	53.2	62.8	63.2	62.3	0.600	45.8	47.2	44.4	0.064	< 0.001*
Know the hazards of hypertension	74.5	84.0	82.8	85.2	0.068	67.1	65.7	68.5	0.058	< 0.001*
know the risk factors of hypertension	79.4	88.8	87.2	90.4	0.003*	72.0	72.3	71.8	0.707	< 0.001*
Know less than 6 g salt per day	38.9	48.6	46.0	51.3	0.002*	31.3	31.4	31.2	0.899	< 0.001*
Know that eating less salt lowers blood pressure	65.7	69.8	65.8	73.8	< 0.001*	62.5	60.1	64.9	0.001*	< 0.001*
Know the hazards of high salt	71.1	80.5	77.5	83.5	< 0.001*	63.7	61.0	66.4	< 0.001*	< 0.001*
Know what kind of people should eat a low-salt diet	79.3	87.6	84.9	90.3	< 0.001*	72.9	71.5	74.3	0.040*	< 0.001*
Know how to use salt-restriction spoon correctly	8.8	11.3	10.7	11.9	0.298	6.8	7.0	6.6	0.645	< 0.001*
Know low-sodium salt	30.0	42.6	37.4	47.6	< 0.001*	20.2	19.5	20.9	0.259	< 0.001*
Know that low-sodium salt helps control blood pressure	20.8	29.0	24.8	33.2	< 0.001*	14.4	13.3	15.4	0.048*	< 0.001*
**Attitude**										
Evaluate whether low-salt diet affects taste of food										
Great influence	11.6	11.2	13.8	8.7	< 0.001*	12.0	14.2	9.8	< 0.004*	0.013*
Has some influence, but can accept	62.8	65.5	64.5	66.3		60.8	59.3	62.3		
No effect	25.5	23.4	21.7	25.0		27.3	26.6	27.9		
Approve that low-salt diet should be promoted among the crowd	87.1	88.5	86.3	90.6	< 0.001*	86.0	83.9	88.2	< 0.001*	0.002*
Approve of low-salt diet	88.2	89.2	86.1	92.2	< 0.001*	87.4	84.1	90.8	< 0.001*	0.018*
Approve of the nutrition labeling of prepackaged food	75.1	79.8	78.8	80.8	0.157	71.5	72.4	70.5	0.162	< 0.001*
Approve that the nutrition labeling of prepackaged food will help to choose low-salt diet	72.6	76.3	73.6	79.0	< 0.001*	69.8	70.1	69.5	0.709	< 0.001*
**Behavior**										
Self-assessment salt level										
Not much	28.4	28.3	26.0	30.6	< 0.001*	28.5	27.1	29.9	0.030*	0.644
Moderate	52.9	53.6	53.9	53.2		52.4	53.0	51.8		
Excessive	18.6	18.1	20.1	16.1		19.1	19.9	18.2		
Received publicity or education on low-salt diet	49.0	51.8	46.3	57.3	< 0.001*	46.7	43.2	50.3	< 0.001*	< 0.001*
Once promoted low-salt diet knowledge to people around	69.0	72.1	68.0	75.3	< 0.001*	66.4	60.9	71.1	< 0.001*	< 0.001*
Pay attention to the nutrition label of prepackaged food	22.1	28.2	23.9	32.5	< 0.001*	17.4	16.8	17.9	0.325	< 0.001*
Plan to reduce salt	78.0	80.0	75.7	84.3	< 0.001*	76.5	71.4	81.6	< 0.001*	< 0.001*
Take initiative to reduce salt	58.3	61.3	54.5	68.0	< 0.001*	55.9	48.9	63.0	< 0.001*	< 0.001*
Using or used salt-restriction spoon	12.0	14.5	15.0	14.0	0.399	10.0	10.4	9.5	0.321	< 0.001*
Using salt-restriction spoon correctly	5.6	7.5	7.4	7.5	0.948	4.1	4.4	3.7	0.251	< 0.001*
Using or used low-sodium salt	15.7	24.5	20.6	28.4	< 0.001*	8.7	8.2	9.3	0.179	< 0.001*

^a^Significant difference between male and female in the KAB rates.

^b^Significant difference between urban and rural in the KAB rates.

*P < 0.05.

**Table 3:** Knowledge, attitudes, and behaviors related to salt among different hypertensive status, SRHPP 2017–2018: %.

	All objects (n = 7512)	Optimal (n = 2194)	Prehypertensive (n = 2665)	Hypertensive (n = 2653)	*P* value for trend
**Knowledge**					
Know the diagnostic criteria of hypertension	53.2	52.4	52.1	55.1	0.062
Know the hazards of hypertension	74.5	77.7	74.8	71.7	< 0.001*
know the risk factors of hypertension	79.4	84.8	79.2	75.2	< 0.001*
Know less than 6 g salt per day	38.9	43.6	39.8	34.2	< 0.001*
Know that eating less salt lowers blood pressure	65.7	66.0	63.3	67.8	0.002*
Know the hazards of high salt	71.1	76.2	69.8	68.1	< 0.001*
Know what kind of people should eat a low-salt diet	79.3	83.1	79.5	76.1	< 0.001*
Know how to use salt-restriction spoon correctly	8.8	8.9	8.6	8.8	0.931
Know low-sodium salt	30.0	35.8	29.7	25.6	< 0.001*
Know that low-sodium salt helps control blood pressure	20.8	25.9	20.6	16.7	< 0.001*
**Attitude**					
Evaluate whether low-salt diet affects taste of food					
Great influence	11.6	9.6	11.0	13.9	0.580
Has some influence, but can accept	62.8	67.0	63.0	59.3	
No effect	21.2	19.8	21.6	21.8	
Approve that low-salt diet should be promoted among the crowd	87.1	89.9	86.8	85.1	< 0.001*
Approve of low-salt diet	88.2	89.4	87.2	88.2	0.069
Approve of the nutrition labeling of prepackaged food	75.1	81.2	77.8	67.4	< 0.001*
Approve that the nutrition labeling of prepackaged food will help to choose low-salt diet	72.6	77.9	75.4	65.5	< 0.001*
**Behavior**					
Self-assessment salt level					
Not much	28.4	26.8	27.8	30.4	0.043*
Moderate	52.9	57.1	56.4	46.0	
Excessive	18.6	16.0	15.8	23.6	
Received publicity or education on low-salt diet	49.0	46.1	45.8	54.5	< 0.001*
Once promoted low-salt diet knowledge to people around	69.0	71.6	69.3	66.9	0.044*
Pay attention to the nutrition label of prepackaged food	22.1	25.5	23.7	17.9	< 0.001*
Plan to reduce salt	78.0	77.3	76.9	79.8	0.038*
Take initiative to reduce salt	58.3	57.4	56.2	61.2	< 0.001*
Using or used salt-restriction spoon	12.0	11.6	12.4	11.9	0.700
Using salt-restriction spoon correctly	5.6	5.2	5.4	6.0	0.456
Using or used low-sodium salt	15.7	18.9	15.5	13.2	< 0.001*

*P < 0.05.

### Associations of knowledge, attitudes and behaviors with 24-h urinary sodium and potassium excretion

In the fully adjusted linear regression models, 24-h urinary sodium excretion was inversely associated with knowing the risk factors for hypertension (− 262.93 mg; 95% CI − 483.02 to − 42.83) and positively associated with the self-assessed salt level (276.18 mg; 95% CI 153.66–398.69) (*P* < 0.05). Twenty-four-hour urinary potassium excretion was positively associated with 6 variables related to knowledge and behaviors, and the β-coefficient ranged from 91.43 to 152.92 mg (β-coefficient: know the diagnostic criteria of hypertension, 103.28 mg; know what kind of people should eat a low-salt diet, 152.83 mg; know how to use salt-restricted spoons correctly, 136.53 mg; received publicity or education on a low-salt diet, 106.64 mg; take initiative to reduce salt, 91.43; use low-sodium salt, 152.92 mg; *P* < 0.05). In preliminary adjusted linear regression models, we also observed that almost all KAB-related variables were linearly related to 24-h urinary potassium excretion (*P* < 0.05). The relevant results are presented in Table 4.

**Table 4:** Association (adjusted β-coefficient and 95%CI) between various KAB exposures studied with 24-h urinary sodium and potassium excretion among Zhejiang residents, SRHPP 2017–2018.

	24-h sodium excretion, mg		24-h potassium excretion, mg	
	β-coefficient^a^ (95% CI)		β-coefficient^a^ (95% CI)	
	Adjusted for age, sex, ethnicity	Fully adjusted model^b^	Adjusted for age, sex, ethnicity	Fully adjusted model^b^
**Knowledge**				
Know the diagnostic criteria of hypertension	− 0.43 (− 168.50 to 167.64)	− 7.62 (− 185.69 to 170.45)	187.20 (115.22 to 259.19)*	103.28 (27.06 to 179.50)*
Know the hazards of hypertension	− 148.27 (− 344.23 to 47.68)	− 171.13 (− 376.93 to 34.67)	168.38 (84.15 to 252.61)*	66.80 (− 21.47 to 155.08)
know the risk factors of hypertension	− 213.90 (− 423.24 to − 4.57)*	− 262.93 (− 483.02 to − 42.83)*	168.47 (78.30 to 258.63)*	56.84 (− 37.79 to 151.48)
Know less than 6 g salt per day	− 108.96 (− 281.63 to 63.72)	− 113.62 (− 296.03 to 68.79)	156.55 (82.29 to 230.80)*	68.85 (− 9.48 to 147.19)
Know that eating less salt lowers blood pressure	− 108.61 (− 286.25 to 69.03)	− 132.56 (− 314.15 to 49.03)	102.92 (26.31 to 179.53)*	27.56 (− 50.52 to 105.63)
Know the hazards of high salt	− 91.69 (− 278.88 to 95.50)	− 125.16 (− 322.15 to 71.83)	179.86 (99.50 to 260.23)*	80.34 (− 4.25 to 164.92)
Know what kind of people should eat a low-salt diet	− 148.89 (− 356.50 to 58.72)	− 169.56 (− 385.87 to 46.75)	247.42 (158.43 to 336.42)*	152.83 (60.07 to 245.59)*
Know how to use salt-restriction spoon correctly	− 46.78 (− 357.98 to 264.41)	− 24.45 (− 339.51 to 290.61)	221.07 (86.84 to 355.29)*	136.53 (1.13 to 271.93)*
Know low-sodium salt	− 43.84 (− 224.96 to 137.28)	− 42.37 (− 233.52 to 148.78)	172.47 (94.71 to 250.22)*	79.46 (− 2.53 to 161.46)
Know that low-sodium salt helps control blood pressure	− 42.15 (− 243.63 to 159.33)	− 50.71 (− 261.98 to 160.56)	184.59 (98.03 to 271.16)*	86.01 (− 4.62 to 176.65)
**Attitude**				
Evaluate whether low-salt diet affects taste of food	− 25.99 (− 164.77 to 112.78)	0.13 (− 137.37 to 137.62)	5.29 (− 54.71 to 65.29)	7.66 (− 51.45 to 66.77)
Approve that low-salt diet should be promoted among the crowd	− 88.97 (− 344.20 to 166.26)	− 61.10 (− 317.62 to 195.42)	142.20 (31.98 to 252.43)*	73.61 (− 36.69 to 183.91)
Approve of low-salt diet	− 209.37 (− 470.52 to 51.77)	− 204.65 (− 465.87 to 56.57)	153.24 (40.41 to 266.07)*	89.51 (− 22.86 to 201.88)
Approve of the nutrition labeling of prepackaged food	− 30.63 (− 227.84 to 166.58)	− 41.35 (− 242.68 to 159.99)	138.24 (53.22 to 223.26)*	61.18 (− 25.34 to 147.70)
Approve that the nutrition labeling of prepackaged food will help to choose low-salt diet	− 136.41 (− 326.80 to 53.97)	− 147.26 (− 341.21 to 46.69)	125.70 (43.55 to 207.85)*	55.68 (− 27.72 to 139.07)
**Behavior**				
Self-assessment salt level	304.23 (181.22 to 427.24)*	276.18 (153.66 to 398.69)*	− 24.39 (− 77.96 to 29.19)	− 14.22 (− 67.22 to 38.77)
Received publicity or education on low-salt diet	135.04 (− 32.44 to 302.53)	135.25 (− 32.42 to 302.93)	145.62 (73.58 to 217.66)*	106.64 (34.81 to 178.47)*
Once promoted low-salt diet knowledge to people around	5.40 (− 251.52 to 262.32)	− 6.17 (− 263.49 to 251.15)	153.37 (42.10 to 264.65)*	104.61 (− 6.85 to 216.07)
Pay attention to the nutrition label of prepackaged food	140.73 (− 62.37 to 343.84)	154.50 (− 54.69 to 363.69)	166.15 (78.74 to 253.57)*	80.32 (− 9.51 to 170.15)
Plan to reduce salt	51.49 (− 150.39 to 253.37)	50.14 (− 151.86 to 252.15)	81.57 (− 5.59 to 168.73)	41.15 (− 45.61 to 127.92)
Take initiative to reduce salt	10.10 (− 162.81 to 183.02)	29.06 (− 144.71 to 202.84)	128.20 (53.76 to 202.64)*	91.43 (16.93 to 165.94)*
Using or used salt-restriction spoon	44.82 (− 223.84 to 313.47)	− 16.74 (− 287.68 to 254.19)	155.39 (39.39 to 271.39)*	97.97 (− 18.51 to 214.45)
Using salt-restriction spoon correctly	31.11 (− 345.51 to 407.74)	20.04 (− 358.60 to 398.67)	244.01 (81.47 to 406.55)*	158.40 (− 4.35 to 321.15)
Using or used low-sodium salt	89.89 (− 142.63 to 322.40)	123.83 (− 115.64 to 363.29)	240.93 (141.23 to 340.64)*	152.92 (50.29 to 255.54)*

*CI* confidence interval.

^a^β-coefficients for KAB indicate change in mg of 24-h urinary sodium or potassium excretion associated with change of options (from "No" to "Yes").

^b^Fully adjusted models included age, sex, ethnicity, education, body mass index, history of cardiovascular disease, diabetes status, chronic kidney disease, smoking status, alcohol use status and physical activity.

*P < 0.05.

### Associations of knowledge, attitudes and behaviors with hypertensive status

Mastering more salt-related knowledge and taking active salt reduction measures were significantly inversely associated with the occurrence of hypertension in the optimal blood pressure and prehypertension group. For example, in the fully adjusted multivariable logistic model, for the optimal blood pressure, knowing the diagnostic criteria of hypertension in comparison with not knowing had 32% lower odds of hypertension (OR, 0.68; 95% CI 0.59–0.79) and prehypertension, which decreased by 25% (OR, 0.75; 95% CI 0.66–0.85). Similar results mainly appeared for 5 knowledge-related variables (odds ratio from 0.68 to 0.83), 1 belief-related variable (odds ratio 0.81) and 4 behavior-related variables (odds ratio from 0.65 to 0.86). The results of this study confirmed that even in the prehypertension stage, receiving health education (OR, 0.84; 95% CI 0.74–0.95), planning to reduce salt consumption (OR, 0.79; 95% CI 0.68–0.91), and taking action (OR, 0.86; 95% CI 0.76–0.97) were significantly inversely associated with the development of hypertension (*P* < 0.05). The relevant results are presented in Table 5.

**Table 5:** Association (adjusted odds ratio and 95%CI) between various KAB exposures studied with hypertensive status among Zhejiang residents, SRHPP 2017–2018.

	Hypertensive^b^ (n = 2653)	Optimal (n = 2194)		Prehypertensive (n = 2665)	
		OR, 95% CI		OR, 95% CI	
		Adjusted for age, sex, ethnicity	Fully adjusted model^a^	Adjusted for age, sex, ethnicity	Fully adjusted model^a^
**Knowledge**					
Know the diagnostic criteria of hypertension	1.0	0.70 (0.62–0.80)*	0.68 (0.59–0.79)*	0.76 (0.67–0.85)*	0.75 (0.66–0.85)*
Know the hazards of hypertension	1.0	0.77 (0.66–0.90)*	0.74 (0.62–0.87)*	0.85 (0.75–0.97)*	0.83 (0.72–0.96)*
know the risk factors of hypertension	1.0	0.83 (0.70–0.98)*	0.81 (0.67–0.97)*	0.80 (0.70–0.92)*	0.80 (0.68–0.93)*
Know less than 6 g salt per day	1.0	1.04 (0.91–1.19)	0.99 (0.85–1.16)	1.04 (0.93–1.18)	1.02 (0.89–1.16)
Know that eating less salt lowers blood pressure	1.0	0.69 (0.60–0.79)*	0.70 (0.60–0.82)*	0.72 (0.64–0.81)*	0.72 (0.64–0.82)*
Know the hazards of high salt	1.0	0.83 (0.72–0.96)*	0.81 (0.69–0.96)*	0.79 (0.70–0.90)*	0.78 (0.68–0.90)*
Know what kind of people should eat a low-salt diet	1.0	0.78 (0.66–0.92)*	0.78 (0.65–0.93)*	0.85 (0.74–0.98)*	0.86 (0.74–1.01)
Know how to use salt-restriction spoon correctly	1.0	0.86 (0.68–1.08)	0.88 (0.69–1.13)	0.87 (0.71–1.07)	0.88 (0.71–1.10)
Know low-sodium salt	1.0	1.08 (0.93–1.24)	1.05 (0.89–1.24)	0.98 (0.86–1.12)	0.97 (0.84–1.12)
Know that low-sodium salt helps control blood pressure	1.0	1.15 (0.98–1.35)	1.09 (0.91–1.31)	1.03 (0.89–1.20)	0.99 (0.84–1.17)
**Attitude**					
Evaluate whether low-salt diet affects taste of food					
Great influence	1.0	0.91 (0.72–1.15)	0.93 (0.72–1.19)	0.83 (0.68–1.01)	0.82 (0.68–1.02)
Has some influence, but can accept	1.0	0.96 (0.82–1.12)	0.90 (0.76–1.06)	0.91 (0.79–1.04)	0.88 (0.76–1.01)
No effect^c^		1.00	1.00	1.00	1.00
Approve that low-salt diet should be promoted among the crowd	1.0	0.98 (0.81–1.20)	0.99 (0.80–1.23)	0.95 (0.81–1.12)	0.96 (0.80–1.14)
Approve of low-salt diet	1.0	0.78 (0.64–0.96)*	0.78 (0.63–0.98)*	0.81 (0.68–0.96)*	0.81 (0.67–0.97)*
Approve of the nutrition labeling of prepackaged food	1.0	0.97 (0.83–1.14)	0.95 (0.80–1.13)	1.10 (0.96–1.26)	1.08 (0.94–1.24)
Approve that the nutrition labeling of prepackaged food will help to choose low-salt diet	1.0	0.97 (0.83–1.12)	0.91 (0.77–1.07)	1.13 (0.99–1.28)	1.09 (0.95–1.25)
**Behavior**					
Self-assessment salt level					
Not much	1.0	1.18 (0.97–1.43)	1.05 (0.86–1.29)	1.35 (1.14–1.59)*	1.28 (1.08–1.53)*
Moderate	1.0	1.21 (1.02–1.44)*	1.15 (0.95–1.39)	1.47 (1.26–1.71)*	1.44 (1.23–1.69)*
Excessive^c^		1.0	1.0	1.0	1.0
Received publicity or education on low-salt diet	1.0	0.84 (0.73–0.95)*	0.84 (0.73–0.97)*	0.84 (0.75–0.94)*	0.84 (0.74–0.95)*
Once promoted low-salt diet knowledge to people around	1.0	0.91 (0.74–1.12)	0.85 (0.68–1.06)	0.98 (0.82–1.17)	0.94 (0.78–1.13)
Pay attention to the nutrition label of prepackaged food	1.0	1.07 (0.91–1.25)	1.11 (0.93–1.32)	1.14 (0.99–1.31)	1.16 (0.99–1.35)
Plan to reduce salt	1.0	0.62 (0.53–0.73)*	0.65 (0.55–0.77)*	0.76 (0.66–0.88)*	0.79 (0.68–0.91)*
Take initiative to reduce salt	1.0	0.79 (0.69–0.90)*	0.82 (0.71–0.95)*	0.83 (0.74–0.93)*	0.86 (0.76–0.97)*
Using or used salt-restriction spoon	1.0	0.84 (0.69–1.03)	0.89 (0.71–1.10)	0.93 (0.78–1.11)	0.96 (0.80–1.15)
Using salt-restriction spoon correctly	1.0	0.78 (0.59–1.04)	0.84 (0.62–1.14)	0.83 (0.65–1.06)	0.84 (0.65–1.09)
Using or used low-sodium salt	1.0	1.16 (0.97–1.38)	1.14 (0.93–1.39)	1.06 (0.90–1.25)	1.05 (0.87–1.25)

*CI* confidence interval, *OR* odds ratio.

^a^Fully adjusted models included age, sex, ethnicity, education, body mass index, history of cardiovascular disease, diabetes status, chronic kidney disease, smoking status, alcohol use status and physical activity.

^b^With hypertensive as a reference.

^c^With this row as a reference.

*P < 0.05.

## Discussion

Hypertension is the leading modifiable risk factor for CVDs, which is the primary cause of death in China^32,33^. The prevalence of hypertension increased from 18.0% to 23.2% from 2002 to 2015^34^. This survey found that the proportion of the population with prehypertension was nearly 36%, which is consistent with the latest China Hypertension Survey in 2015 showing that approximately 244.5 million individuals (23.2%) of the Chinese adult population had hypertension, whereas approximately 435.3 million individuals (41.3%) had prehypertension according to the Chinese guidelines^35^.

This is a large-scale population study with local representativeness. For the first time in China, the association between KAB and hypertensive status categories and the linear association between KAB and 24-h urinary sodium and potassium excretion were studied. An important advantage of this study is that it carried out 24-h urine collection and urinary sodium and potassium tests, which are used to accurately assess sodium intake at the population level. The main source of sodium intake in the Chinese population is family cooking, accounting for approximately 80%^36^. At the same time, Chinese cooking methods are relatively complicated, and there are many sodium-containing condiments and foods, such as soy sources and monosodium glutamate, pickles, plums, instant noodles, and biscuits^34^. Multiple 24-h urinary sodium excretion measurements is considered the gold standard for estimated sodium intake^30,38,39^. Therefore, the results of our association study are more reliable than the results obtained from estimating dietary sodium^40^. This study collected the variables of the KAB related to salt and developed a standardization questionnaire, which basically covers the salt use of the Chinese population, even though it has not undergone rigorous reliability and validity analysis. Compared with similar research^16,41,42^, our topic setting is more in-depth and detailed.

In this study, urban and rural areas classified according to household registration implied participants’ educational attainment, family income, and health expenditure support. Higher classes would afford and adopt healthier diet practices^43^. The results of this study showed that salt-related KAB in rural areas was significantly lower than that in urban areas. Rural residents have a low education level, have low access to public health and medical care, and maintain unhealthy concepts and lifestyles (such as incorrectly recognizing that less sodium affects physical strength, more sodium causes no harm, or routinely eating high-salt pickled foods). Therefore, the focus of the health education campaign in rural areas is that knowledge is easy to understand and accept, and the risks of salt should be explained. In addition, women, generally the head chefs of home cooking and the gatekeepers to diet and health in China^44^, are an important group to target with the educational initiative. Women’s demands for their own health and the maintenance of a healthy lifestyle far exceed those of men. One of the important reasons for the success of UK salt reduction is the targeting of women consumers on the relationship between sodium and health^45^. Hence, special attention should be given to women, especially in rural areas.

People need to know their sodium and potassium intake levels and the gap between the recommended amounts, as a basic reference value and as an intrinsic motivation to enhance their salt-reduction awareness and to stimulate salt-reduction behaviors. The roughest way is a consumer self-assessment. However, different sexes, ages, education levels, and hypertensive statuses will appear to have varying degrees of inaccuracy in self-reported low sodium diets among Chinese individuals^46^. This study found that 81.4% of the participants reported that they ate low or medium levels of salt, while only a very small proportion actually achieved the 6 g/day target. People generally underestimate their salt intake, which highlights an opportunity for interventions that can translate that intent into reality. In the linear regression relationship study, it was indeed found that self-assessed salt contributed to 24-h urinary sodium (β-coefficient, 276.18 mg/d; 95% CI 153.66–398.69). This suggests that consumers should start to reduce salt when they realize that they eat too much salt.

In addition, although potassium supplementation is limited by the applicable population, the emphasis on potassium supplementation should be increased to the same level as sodium reduction. A systematic review and meta-analysis showed that only salt substitutes were effective in lowering BP (significantly reduced systolic BP by 5.7 mm Hg and diastolic BP by 2.0 mm Hg) among the many salt reduction strategies (including health education, salt restriction diet, salt-restriction spoons and salt substitutes)^47^. Compared with the adjusted multivariable linear regression model of sodium and potassium, it was found that KAB has a smaller effect on sodium but a greater effect on potassium, which is enough to make us feel hopeful. This showed that KAB is necessary for potassium supplementation, and we should re-examine the effect of KAB on urinary electrolytes.

The Health Belief Model (HBM) postulates that after the individual fully recognizes the disease and the cause, as long as they gain knowledge and skills, they believe that taking preventive measures will allow them to obtain health benefits and are more likely to take action^48^. More salt reduction knowledge and stronger salt reduction awareness and beliefs will produce more profound and autonomous salt reduction behavior, and the three form a closed-loop logical chain.

Our research found that the knowledge and belief indicators among those with optimal blood pressure are better than those of pre-hypertensive and hypertensive patients. According to the logic, their salt-reduction behavior should be better than that of hypertensive patients. However, the results of the investigation are the opposite. The cross-sectional survey design concealed any cause and effect relationship, and due to the high intensity of engagement with health care providers, one-on-one interactions, or small group activities, hypertensive patients had relatively more salt reduction behavior.

Social cognitive theory (SCT) suggests that people need mastery of both knowledge and skills to perform a given behavior^49^. In practical applications, the spread of knowledge is often overemphasized, and the importance of belief is ignored, which means that most campaigns focus on raising knowledge, which may not always lead to action. One of the barriers to progress in salt reduction among the general population is that current strategies to reduce salt intake have not raised individuals’ interest and beliefs in engaging in salt reduction^50^. When the participants were surveyed on salt reduction awareness and belief, they tended to approve and support, which is likely because social approval bias results in more socially desirable responses^51^. However, in the multivariable logistic regression analysis of KAB and hypertension, we found that regardless of prehypertension or optimal blood pressure, knowledge and behaviors play an important role in preventing the development of hypertension, while beliefs, as the “bridge” between knowledge and behaviors, seemed to have no effect, which again reminds us that we should strengthen the individual's awareness and belief in salt reduction. The results of this study confirmed that even in the prehypertension stage, mastering most of the knowledge related to salt, realizing the importance of salt reduction, planning and taking positive actions can all resist the development of hypertension. Therefore, earlier cognition, earlier belief, and earlier implementation are the correct ways to reduce salt.

Salt-restricted spoons are spoons with gram scales (e.g., 1 g, 2 g, 6 g) that are being pushed by China Healthy Lifestyles for All Action Spoons^52^ and have been shown to effectively lower blood pressure^53^. However, our survey pointed out that the adoption rate (12.0%) of salt-restricted spoons in China is very low, followed by a lower correct awareness rate (8.8%) and correct usage rate (5.6%). The findings of this study suggested that salt-restricted spoons had little effect on sodium intake and blood pressure control. We should popularize their correct usage to distinguish them from ordinary spoons. Furthermore, a recent modeling study reported that nationwide implementation of potassium-enriched salt substitution (20–30% potassium chloride) or low-sodium salt in China was estimated to result in a substantial net benefit, preventing approximately one in nine deaths from CVDs overall^9^. Similar to the salt-restricted spoon, the awareness rate, benefit awareness rate and adoption rate of low-sodium salt (30.0%, 20.8%, and 15.7%, respectively) were very low, which may be related to its low shelf rate and high sale price in supermarkets or stores. It is not a new thing. Our research showed that low-sodium salt had its main effect on potassium supplementation, and it was indeed worthy of application and promotion.

We found that three-fourths of participants supported nutrition labeling and believed nutrition labeling of prepackaged food will help to choose a low-salt diet. However, very few residents (22.1%) reported reading nutrition labels while purchasing food^54^. There are many types of salt labels for prepackaged foods, including food labels, color-coded labeling (e.g., traffic light labels in the UK), warning labels (e.g., ‘high in:::’ warning statements in South America and the Health Star Rating system in Australia)^55,56^. At present, there is a publicity of front-of-pack labeling to make consumers pay more attention to the consumption of salt^57^. In China, some salt condiments have been labeled "low-salt soy sauce (salt content reduced by 25%)" and "low-salt pickles", and some salt packages have been printed with the slogan "6 g salt per person per day". This is an improvement and an important step from within the food industry. Therefore, consumers should be guided to correctly identify high-salt foods, be alert to "hidden" salt, and try to choose low-salt foods with proper use of nutrition labeling.

Our findings are subject to the following limitations. First, this study only collected and tested 24-h urine once because of the associated high participant burden and high cost^58^. Given that the large within-individual variability in consumption will have been overestimated, the strengths of the associations of sodium and potassium intake with the various KAB exposures studied will probably have been underestimated^59^. Second, because the exposed variables of the study were knowledge, beliefs and behaviors from the respondents' self-report, participants answered that options tended to be positive or sounded more in line with health concepts, leading to overestimation or underestimation in the association research. Third, this study is based on a cross-sectional survey and remains uncertain about cause and effect in outcomes and exposures, meaning that causality cannot be inferred from these data.

## Conclusion

There is much room for improvement of salt-related KAB in the Chinese population, especially in rural areas, among men and among hypertensive patients. This study is the first large-scale study of KAB and 24-h urinary sodium, potassium excretion and hypertensive status in China, and its results will be valuable for policy-makers to develop and implement public health strategies. Strong evidence of the association between them suggests that modifying the population levels of these indicators of KAB might be an effective way of reducing sodium and supplementing potassium in China. Mastering more knowledge, enhancing salt-reduction awareness and more active salt-reduction behavior can help to suppress the transition from normal blood pressure to hypertension. This study provides important evidence and insight into China’s efforts to meet the targets of salt reduction and hypertension prevention.

## Supplementary Information

Supplementary Information.

## Data Availability

The datasets analyzed during the current study are not publicly available because of intellectual property rights but are available from the corresponding author on reasonable request.

## Abbreviations

CVD: Cardiovascular diseases
CDC: Centers for disease control and prevention
SRHPP: Salt reduction and hypertension prevention project
KAB: Knowledge, attitudes and behaviors
HBM: Health belief model
SCT: Social cognitive theory
SBP: Systolic blood pressure
DBP: Diastolic blood pressure
WHO/PAHO: World Health Organization/Pan American Health Organization
BMI: Body mass index
SD: Standard deviation
CI: Confidence interval
OR: Odds ratio
