# Supplementary material for: The association of knowledge, attitudes and behaviors related to salt with 24-h urinary sodium, potassium excretion and hypertensive status
Source: Sci Rep. 2022 Aug 16;12:13901. doi: 10.1038/s41598-022-18087-x (PMC9381520; doi:10.1038/s41598-022-18087-x)
Supplement: Supplementary file 1 — Supplementary Information. [file 41598_2022_18087_MOESM1_ESM.docx]

**Online Supplemental Material**

**The association of knowledge, attitudes and behaviors related to salt with 24-hour urinary sodium, potassium excretion and hypertensive status**

Supplemental Figure1. Geographical distribution of participating counties and cities, SRHPP in Zhejiang Province of China in 2017-2018.


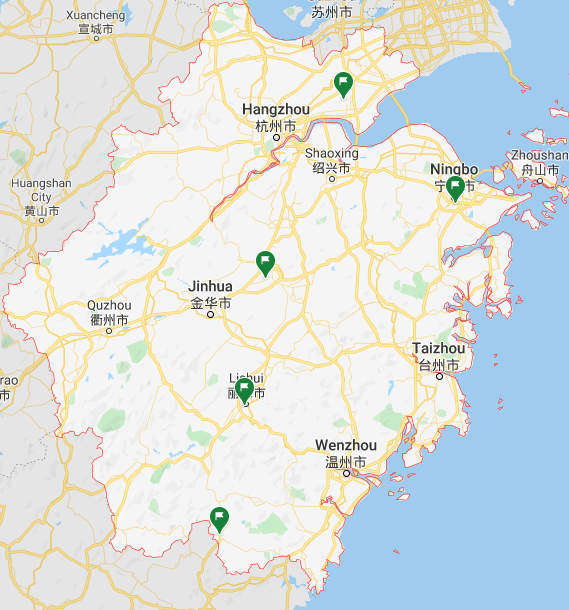

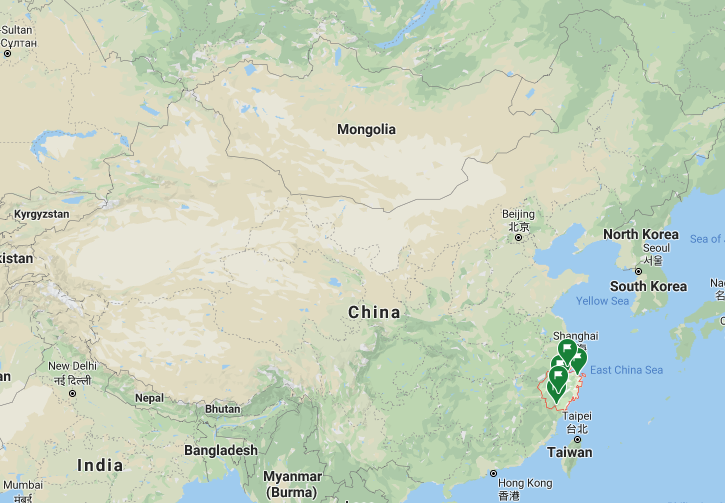
Notes:

© OpenStreetMap contributors. Image credits OpenStreetMap and its contributors. URL: <https://www.openstreetmap.org/copyright.> The picture is edited on Baidu Online Map. URL: <https://map.baidu.com.> The five marked points in the figure represent Yiwu City, Haining City, Taishun County, Yinzhou District, and Liandu District respectively.

Supplemental Table1. Questionnaire about knowledge, attitudes and behaviors related to salt, SRHPP 2017-2018.

| 1. Do you know how much blood pressure in adults can be diagnosed as hypertension? | |
| --- | --- |
|  | (1) 140/90mmHg |
|  | (2) 130/80 mmHg |
|  | (3) 120/80 mmHg |
|  | (9) I don’t know |
| 2. Do you know what diseases hypertension can cause (multiple choices)? | |
|  | (1) Stroke |
|  | (2) Coronary heart disease |
|  | (3) Kidney disease |
|  | (4) Hypertensive heart disease |
|  | (5) Eye diseases |
|  | (9) I don’t know |
| 3. Do you know which factors are related to the prevalence of hypertension (multiple choices)? | |
|  | (1) Overweight or obese |
|  | (2) Long-term excessive drinking |
|  | (3) Long-term high-salt diet |
|  | (4) Have a family genetic history |
|  | (5) Hyperglycemia and hyperlipidemia |
|  | (6) Age |
|  | (7) Stress |
|  | (9) I don’t know |
| 4. Do you know that adults should not eat more than a few grams of salt per person per day? | |
|  | (1) 2g |
|  | (2) 6g |
|  | (3) 9g |
|  | (4) 12g |
|  | (9) I don’t know |
| 5. Do you know that eating less salt helps lower blood pressure? | |
|  | (1) Know |
|  | (2) I don’t know |
| 6. Do you know what diseases may result from eating too much salt (multiple choices)? | |
|  | (1) Hypertension |
|  | (2) Stroke |
|  | (3) Myocardial infarction |
|  | (4) Kidney disease |
|  | (5) Gastric cancer |
|  | (6) Osteoporosis |
|  | (9) I don’t know |
| 7. Do you think you eat too much salt? | |
|  | (1) Not much |
|  | (2) Moderate |
|  | (3) Too much |
| 8. Do you plan to eat less salt after knowing the dangers of eating too much salt? | |
|  | (1) Intend to |
|  | (2) Not intend to |
| 9. Has your family ever used a salt-restriction spoon? | |
|  | (1) Used |
|  | (2) Not used |
| 10. Does your family know how to use salt-restriction spoon? | |
|  | (1) Know |
|  | (2) I don’t know |
| 11. Can your family use the salt-restriction spoon correctly according to the requirements? | |
|  | (1) Yes |
|  | (2) No |
| 12. Do you think low-salt diet should be promoted among the crowd? | |
|  | (1) Should |
|  | (2) Should not |
| 13. Have you ever been promoted or educated on a low-salt diet? | |
|  | (1) Accepted |
|  | (2) Not accepted |
| 14. Have you ever promoted the knowledge of low-salt diet to the people around you? | |
|  | (1) Promoted |
|  | (2) Not promoted |
| 15. Do you think a low-salt diet affects the taste of food? | |
|  | (1) Great influence |
|  | (2) Has a certain influence, but can accept |
|  | (3) No effect |
| 16. What kind of people do you think should pay special attention to low-salt diet (multiple choices)? | |
|  | (1) Hypertensive patients |
|  | (2) Patients with stroke |
|  | (3) Patients with coronary heart disease |
|  | (9) I don’t know |
| 17. Have you paid attention to the salt / sodium content of the food when purchasing processed food? | |
|  | (1) Followed |
|  | (2) Not followed |
| 18. Do you think processed foods should be labeled with the salt / sodium content of the product? | |
|  | (1) Should |
|  | (2) Should not |
| 19. Do you think that labeling the salt / sodium content of processed foods will help you choose low-salt foods? | |
|  | (1) Yes |
|  | (2) No |
| 20. What is your attitude towards a low-salt diet? | |
|  | (1) For |
|  | (2) Against |
| 21. Have you taken active salt reduction measures yourself? | |
|  | (1) Yes |
|  | (2) No |
| 22. Have you heard of low-sodium salt (alternative salt)? | |
|  | (1) Yes |
|  | (2) No |
| 23. Do you know that low-sodium salt helps control blood pressure compared to regular table salt? | |
|  | (1) Know |
|  | (2) I don’t know |
| 24. Have you used low-sodium salt? | |
|  | (1) Used |
|  | (2) Not used |
